# Predicting head and neck cancer response to radiotherapy using mathematical modeling of MRI-based habitats

**DOI:** 10.1038/s41698-026-01344-x

**Published:** 2026-04-16

**Authors:** David A. Hormuth, Michael J. Dubec, Abhishek Rao, Alexandra Lozano Reyes, Kevin J. Harrington, David L. Buckley, James PB O’Connor, Thomas E. Yankeelov

**Affiliations:** 1 Oden Institute for Computational Engineering and Sciences, Austin, TX USA; 2 UT Austin Cancer Research Center The University of Texas at Austin, Austin, TX USA; 3 Division of Cancer Sciences, University of Manchester, Manchester, United Kingdom; 4 Christie Medical Physics and Engineering, The Christie NHS Foundation Trust, Manchester, United Kingdom; 5 Biomedical Engineering, Austin, TX USA; 6 Division of Radiotherapy and Imaging, The Institute of Cancer Research, London, United Kingdom; 7 Biomedical Imaging, University of Leeds, Leeds, United Kingdom; 8 Radiology, The Christie NHS Foundation Trust, Manchester, United Kingdom; 9 Diagnostic Medicine, Austin, TX USA; 10 Imaging Physics MD Anderson Cancer Center, Houston, TX USA

**Keywords:** Cancer, Medical research, Oncology

## Abstract

Accurately predicting hypoxia may enable personalized radiotherapy to improve outcomes through biologically guided dose modulation. To predict hypoxia status, we integrate advanced MRI methods—oxygen-enhanced MRI (OE-MRI) for hypoxia, dynamic contrast-enhanced MRI (DCE-MRI) for perfusion and cellularity—with a mathematical model of radiation response. Data were collected before and during radiotherapy for 20 patients with HPV-associated oropharyngeal cancer. MRI data were analyzed to derive parameters describing hypoxia, perfusion, and cellularity, clustering each tumor into four habitats at each time point. The model was calibrated using n-fold cross-validation to determine optimal parameters describing response over weeks 2 and 4 of radiotherapy in primary and nodal disease. Prediction accuracy was evaluated on unseen data using Pearson (PCC) and concordance correlation coefficients (CCC). Predictions for perfused hypoxic primary and nodal tumors showed strong correlation (PCC ranging from 0.74 to 0.77) and agreement (CCC ranging from 0.68 to 0.70). Using MRI-based habitats, the model accurately forecasts patient-specific tumor response, potentially supporting personalized radiotherapy in head and neck cancer.

## Introduction

Human papillomavirus (HPV)-associated oropharyngeal carcinomas are a common cause of head and neck cancer that have seen increasing incidences in higher-income countries^1,2^. Patients with HPV-positive oropharyngeal cancers have a better prognosis than those with HPV-negative disease, often being cured with standard-of-care chemoradiation. However, acute and long-term side effects can significantly impact quality of life. Given the young age of many affected individuals and the high curability of the disease, several efforts^1,3,4^ have focused on de-escalating or de-intensifying therapy to reduce long-term side effects while preserving efficacy. One promising de-escalation strategy involves monitoring tumor hypoxia before and during treatment to identify patients who may benefit from a reduced radiotherapy (RT) dose^3^. Hypoxia is well known to impair the effectiveness of both RT and chemotherapy, contributing to treatment failure^5–7^. Thus, accurately measuring and predicting changes in tumor hypoxia has the potential to fundamentally improve adaptive RT strategies by delivering lower doses to patients with lower levels of hypoxia.

Historically, positron emission tomography (PET) methods have dominated preclinical and clinical approaches to image and measure tumor hypoxia^8^. However, the development of combined magnetic resonance imaging (MRI) linear accelerators (MR Linac)^9^ for radiotherapy treatment planning and delivery has spurred interest in developing MRI-based techniques to assess hypoxia^10,11^ and characterize the tumor microenvironment^12–16^ for treatment adaptation. One such technique is oxygen-enhanced MRI (OE-MRI), wherein subjects undergo a gas challenge—alternating inhalation of medical air (21% O_2_ gas) with 100% O_2_ gas—while *T*_*1*_-weighted images are collected^17^. Dynamic changes in the longitudinal relaxation rate *R*_*1*_ (where *R*_*1*_ = 1/*T*_*1*_) are then analyzed to identify normoxia (significant increases in *R*_*1*_ in response to O_2_) and hypoxia (no significant change in *R*_*1*_ in response to O_2_)^17^ regions which have been validated pre-clinically to ex vivo tissue samples in multiple tumor models^10,18^. More recently, the feasibility and repeatability of OE-MRI have been demonstrated on an MR Linac system^19^. Subsequently, it was applied in a prospective study to monitor radiation-induced changes in hypoxia as early as week 2 of RT (not always with a change in tumor size). Interestingly, the timing of hypoxia changes varied between primary and nodal tumors^20^ suggesting that lesion-specific de-escalation strategies may be required. These results were consistent with a previous study of RT in patients with non-small cell lung cancer^18^.

Habitat imaging^12,14,15^ divides tumors into habitats (or sub-regions) that share similar physiological or biological properties as characterized by the imaging method. In particular, quantitative MRI or PET data can be used to generate habitats that further characterize tumor hypoxia. In MRI, habitat imaging frequently makes use of the data from dynamic contrast-enhanced MRI (DCE-MRI)^21^, which provides information on both perfusion and cellularity, and diffusion-weighted imaging (DWI)^22^, which primarily reflects tissue cellularity. Both preclinical^14,23^ and clinical^24,25^ habitat imaging has shown promise in stratifying subjects and/or predicting treatment outcomes in response to RT, chemotherapy, or combination chemoradiation. In soft tissue sarcomas, this approach has advanced to clinical application, with image-defined habitats guiding dose escalation in a Phase 2 non-randomized single-arm clinical trial^12^. Building on these developments, we now seek to use OE-MRI data to classify tumor habitats based on hypoxia, perfusion, and cellularity, providing biologically informed initialization and constraints for a mathematical model of HPV-positive oropharyngeal carcinoma response to chemoradiation.

Several efforts have linked imaging data with mathematical modeling to forecast patient outcomes to chemoradiation^4,15,26–30^. In head and neck cancer, ref. ^26^ developed an ordinary differential equation (ODE) model of tumor volume dynamics, incorporating a dynamic carrying capacity initialized and constrained by longitudinal tumor volumes measured from X-ray computed tomography. Using this model, locoregional control and disease-free survival were predicted with a sensitivity/specificity of 76%/83% and 68%/85%, respectively. In subsequent work, Zahid et al. applied this model in an in silico trial to evaluate dose personalization, concluding that 77% of the patients could have received RT doses up to 39 Gy lower than the actual administered dose. Belfatto et al.^30^ applied a similar ODE model to characterize prostate tumor response in rats receiving radiotherapy and imaged with oxygen-sensitive MRI techniques^31^. They observed a correlation between the estimated RT sensitivity parameter and changes in signal intensity during the oxygen challenge, which reflected the presence of extensive hypoxia and resistance to RT. However, the small cohort and the large fitting error limit the interpretability of these findings. A novel modeling approach leveraging habitat imaging was applied by ref. ^15^ in a murine model of glioma. They developed a coupled set of ODEs to describe growth and conversion between three distinct tumor habitats, based on variations in tumor perfusion (estimated from DCE-MRI) and cellularity (estimated from DCE-MRI and DWI). Using a training-test set strategy for parameter calibration across the animal cohort, they demonstrated that prediction errors were not significantly higher than calibration errors, supporting the generalizability of the model in describing heterogeneous tumor growth.

Building on recent advances in OE-MRI, habitat imaging, and mathematical modeling, we present a novel coupled ODE describing tumor habitat dynamics and response to chemoradiation in patients with HPV-positive oropharyngeal carcinoma. We first identify four distinct tumor habitats that capture variations in tumor perfusion, cellularity, and hypoxia status for both primary tumor and nodal lesions. Second, patient-specific time courses of habitat volumes are extracted and used to calibrate the coupled ODE system at both the individual and cohort levels. Finally, we quantify predictive accuracy, by calibrating model parameters on a training set of patients and evaluating model performance across primary tumors and nodal lesions on a separate test set.

## Results

### Classification of image-based tumor habitats

**Fig. 1:**
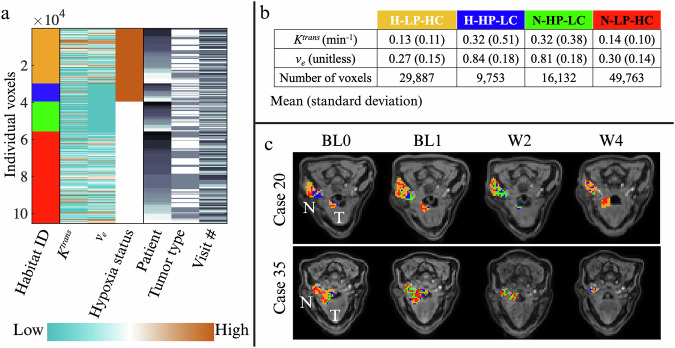
Results of habitat identification. **a** Four habitats were identified *via* clustering on the DCE (*K*^trans^, *v*_*e*_) and OE-MRI (hypoxic or normoxic) data. Patient ID, tumor type (primary or node), and visit number are shown in the heatmap, but were not used for clustering. **b** Cluster centroids (mean and standard deviation) of each parameter are reported and were used to assign biological interpretation. **c** Shows a mapping of these clusters overlaid on the primary (T) and node (N) disease at the two pretreatment visits (BL0 and BL1) and the two on-treatment visits (W2 and W3). For both cases 20 and 35, the nodal disease was larger than the primary tumor.

Figure 1 shows the results of the habitat imaging applied to the entire patient cohort. The classification of each voxel, patient, and time point is shown in Fig. 1a while the centroids of each cluster are reported in Fig. 1b. Figure 1a demonstrates that each habitat consists of a heterogeneous mixture of patient, tumor type and visit number and are then divided by hypoxia status, tissue perfusion (*K*^trans^), and cellularity (defined as 1 - *v*_*e*_). A total of 105,535 voxel values were extracted with the number of voxels per patient ranging from 478 to 12,272; 45.0% (47526/105,535) of the voxels were primary tumor, and 55% of the voxels (58,009/105,535) were nodal lesions. As shown in Fig. 1b, many of the voxels are normoxic (62.4%; 65,895/105,535) and a plurality of the voxels are classified as the N-LP-HC habitat (47.2%; 49,763/105,535). For the hypoxic voxels (37.6%; 39,640/105,535), the H-LP-HC habitat also contained the largest group of hypoxic voxels (28.3%; 29,887/105,535). Visualization of these habitats in two cases mapped to the original anatomical MRI data in Fig. 1c show that both the primary tumor and nodal lesions are highly heterogenous and that the habitats are generally spatially contiguous. Note that in both cases 20 and 35, the nodal lesion is larger than the primary tumor. Additionally, we evaluated the influence of MR system type on habitat centroids (Supplemental Material S4) and observed no significant differences when using the full dataset compared with analyses restricted to MR Linac data alone or diagnostic MR data alone.

### Model calibration (individual and cohort)

**Fig. 2:**
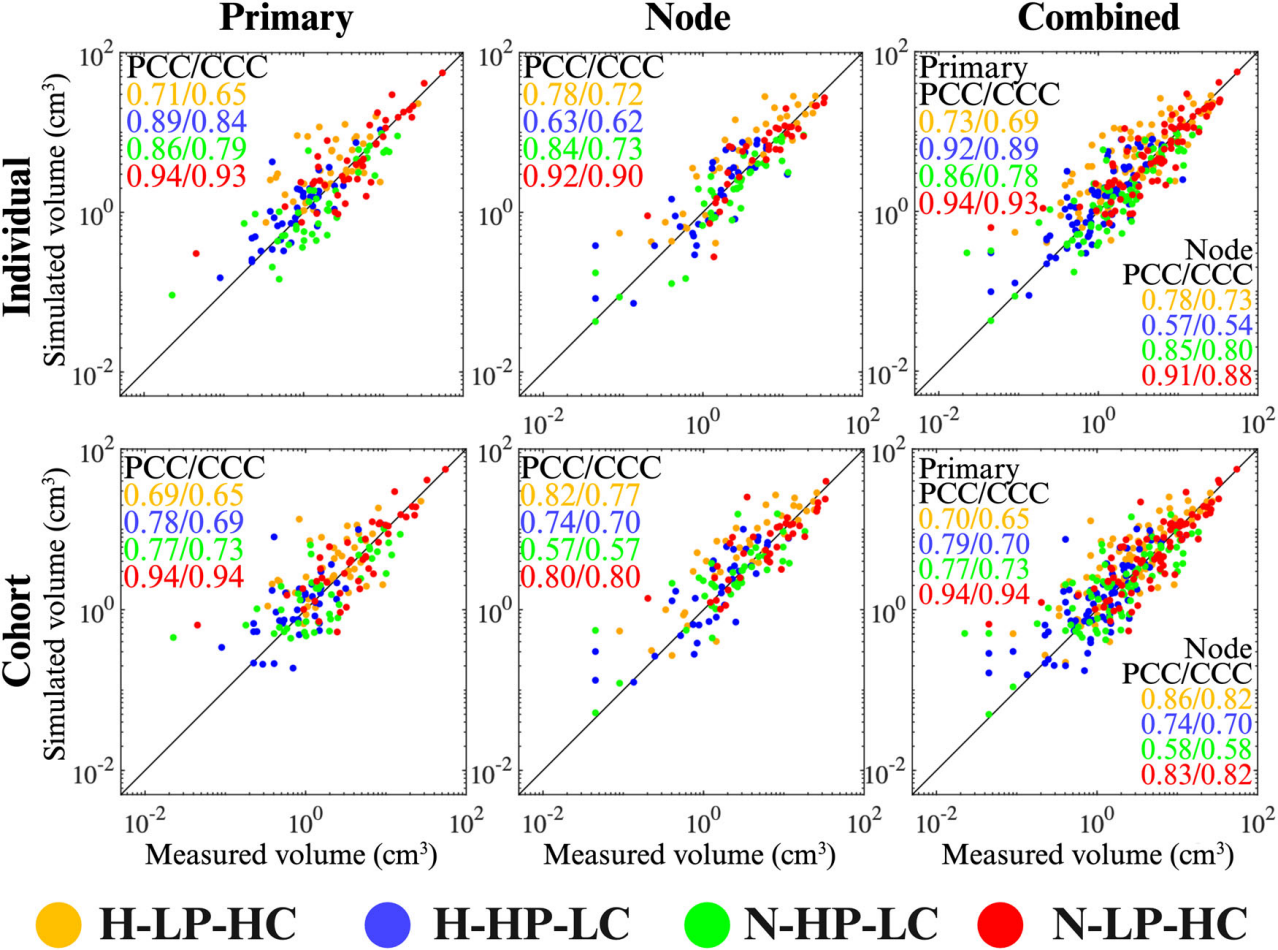
Calibrated versus measured habitat volumes. Rows correspond to individual (top) and cohort (bottom) calibrations; columns show results for primary, nodal, and combined volumes. Each plot displays simulated versus measured habitat volumes across all habitats and time points used for calibration. For the individual calibrations, PCC ranged from 0.57 to 0.94, and CCC ranged from 0.54 to 0.94. For the cohort calibrations, PCCs ranged from 0.57 to 0.94, and CCCs ranged from 0.58 to 0.94. Overall, both PCC and CCC tended to be lower in cohort calibrations compared to individual calibrations.

The results of the two calibration scenarios (individual and cohort) are shown in Fig. 2 and Table 1. The top row plots the simulated (i.e., calibrated) habitat volume versus the measured habitat volume at all of the time points used for model calibration for individual or patient-specific calibration. For the individual calibration, strong correlation and agreement are observed for the primary tumor with PCCs and CCCs ranging from 0.71 to 0.94 and 0.65 to 0.93, respectively, for each patient. For the nodal lesion, lower correlation and agreement was observed with PCCs and CCCs, ranging from 0.63 to 0.92 and 0.62 to 0.90, respectively. Notably, the H-HP-LC habitat volume was poorly characterized for the nodal lesion (PCC/CCC = 0.63/0.62) relative to the primary tumor (0.89/0.84). For the combined calibration, where a single value parameter set was used to describe both primary and node disease dynamics, PCCs and CCCs increased for some habitats (e.g., H-HP-LC for primary) and decreased for others (e.g., H-HP-LC for node). For the cohort calibration (bottom row), PCC and CCCs generally decreased for the primary tumor for both the primary-only and combined calibrations. However, for the nodal lesion the PCC and CCC increased for the H-HP-LC habitat and decreased for the N-HP-LC habitat for both the node only and combined calibrations. Table 1 reports the median, min, and max for each calibrated parameter. Notably, growth rates for the hypoxic compartments (*k*_*1*_ and *k*_*2*_), the transition rate from N-LP-HC to N-HP-LC (*k*_*43*_), and the transition rate and N-LP-HC to H-LP-HC (*k*_*41*_) all had a median value of 0 for individual calibrations and a value of 0 for cohort calibrations. No statistically significant differences were observed between primary and node model parameter values. Supplemental Table 1 reports the median, min, and max for each calibrated parameter, separated by those who received radiotherapy alone or radiotherapy and cisplatin. No statistically significant differences were observed between the two treatment groups.

**Table 1:** Parameter values resulting from the individual and cohort calibrations

	Primary		Node		Combined	
	Individual	Cohort	Individual	Cohort	Individual	Cohort
*k*_*1*_ (day^-1^)	0 [0 0.108]	0	0 [0 0.004]	0	0 [0.108]	0
*k*_*2*_ (day^-1^)	0 [0 0.171]	0	0 [0 0.199]	0	0 [0 0.199]	0
*k*_*3*_ (day^-1^)	0.050 [0 0.593]	0.555	0.061 [0.001 0.518]	0.488	0.095 [0 0.639]	0.882
*k*_*4*_ (day^-1^)	0.011 [0 0.185]	0.128	0.011 [0 0.242]	0.158	0.035 [0 0.242]	0.255
*k*_*12*_ (day^-1^)	0 [0 0.561]	0.232	0 [0 0.522]	0.052	0 [0 0.537]	0.194
*k*_*reoxy*_ (day^-1^)	0.009 [0 0.152]	0.261	0.025 [0 0.101]	0.321	0.044 [0 0.219]	0.450
*k*_*34*_ (day^-1^)	0.248 [0 1.175]	0.614	0.233 [0 1.141]	0.520	0.392 [0 1.200]	0.535
*k*_*43*_ (day^-1^)	0 [0 0.020]	0	0 [0 0.001]	0	0 [0 0.020]	0
*k*_*41*_ (day^-1^)	0 [0 0.541]	0	0 [0 0.014]	0	0 [0 0.541]	0
*α* (Gy^-1^)	0.006 [0 0.036]	0.013	0.007 [0 0.052]	0.009	0.008 [0 0.034]	0.012

For individual calibrations, we report the median [minimum, maximum] values across the cohort.

### Model prediction (cohort)

**Fig. 3:**
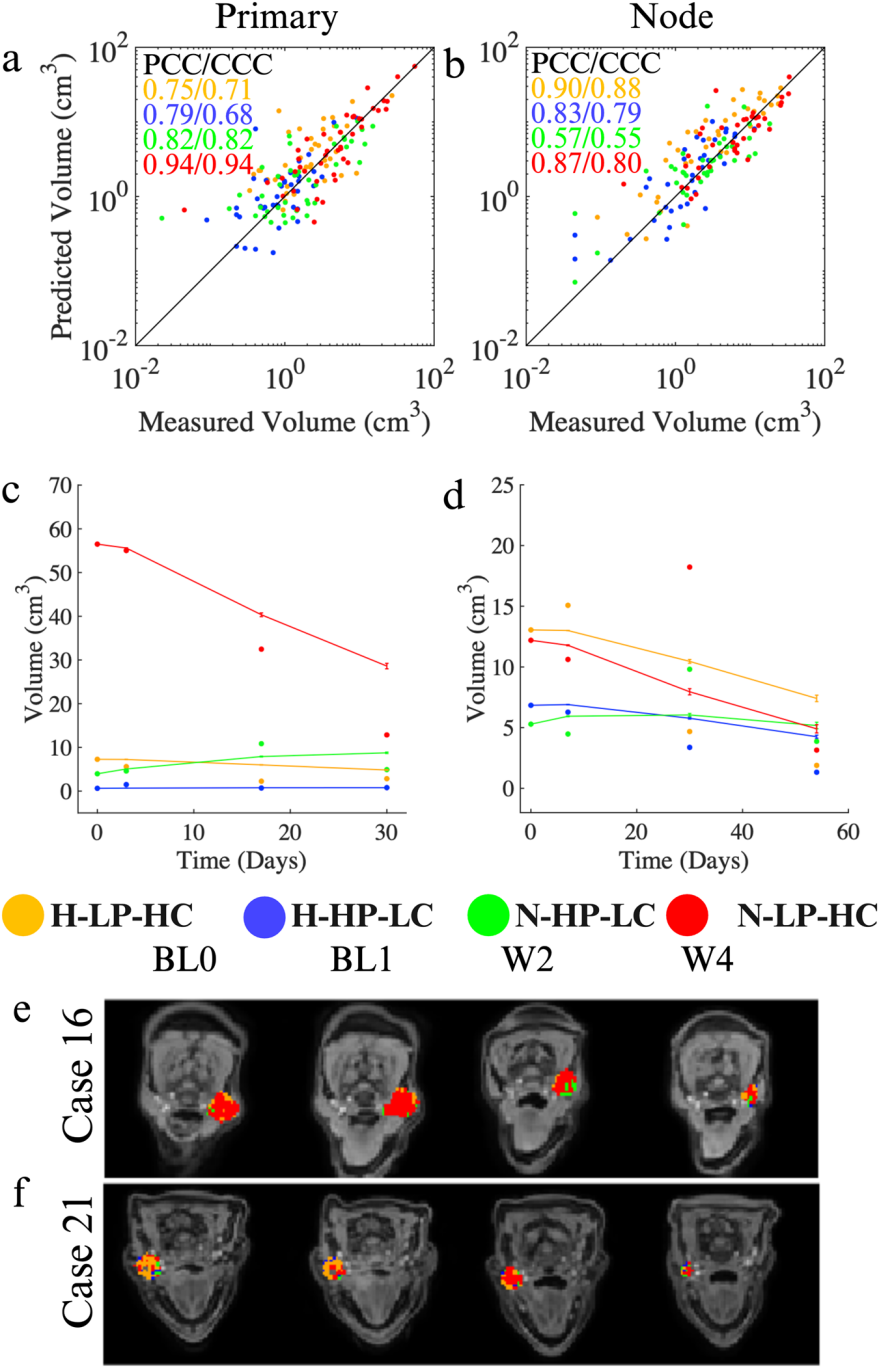
Tumor growth predictions using primary (panels a, c, e) and nodal (panels b, d, f) disease calibrated parameters. **a**, **b** Predicted versus measured habitat volumes for the full cohort. PCC ranged from 0.55 to 0.94, while CCC ranged from 0.55 to 0.94 for both tumor types. **c**, **d** Representative time courses are shown for case 16 (primary tumor) and 21 (nodal tumor). Dots indicate measured values; solid lines and error bars show the model predictions with 95% confidence intervals. **e**, **f** Habitat visualizations before (at baseline 0 and 1; BL0 and BL1) and during treatment (at week 2 and 4 of RT; W2 and W4) for representative patients. In both cases, the central slice reveals a higher proportion of the N-LP-HC habitat compared to others.

**Fig. 4:**
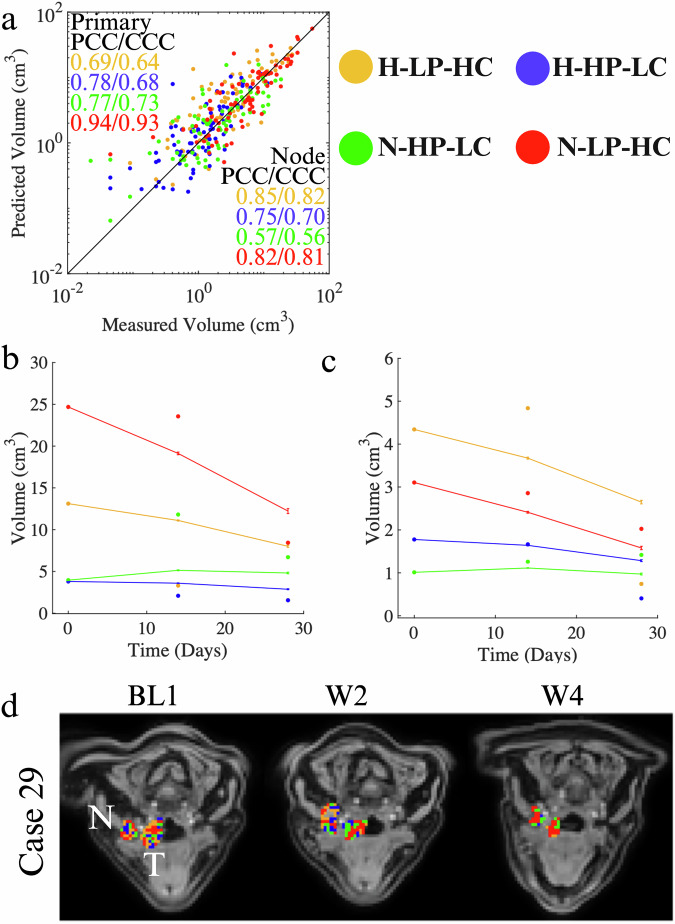
Tumor growth predictions using parameters calibrated on the combined primary and nodal disease. **a** Predicted versus measured habitat volumes for the full cohort. PCC ranged from 0.57 to 0.94, while CCC ranged from 0.56 to 0.93 for both tumor types. **b**, **c** Representative time courses are shown for case 29 (primary, panel **b**; node lesion, panel **c**). Dots indicate measured values; solid lines and error bars show the model predictions with 95% confidence intervals. **d** Habitat visualizations before (baseline 1; BL1) and during treatment (week 2 and 4 of RT; W2 and W4) for a representative patient. In the central slice, a noticeable decrease in H-HP-LC is observed during radiotherapy.

The results of the third calibration scenario (calibration and prediction) are shown in Figs. 3 and 4. Figure 3 shows the results of the primary tumor (Fig. 3a, c, e) and nodal lesion-specific (Fig. 3b, d, f) predictions. For each prediction, only the patient’s initial habitat composition and the parameter set from the training cohort were used to forecast treatment response. For the primary tumor (Fig. 3a), strong correlation and agreement was observed for the H-LP-HC, H-HP-LC, and N-LP-HC habitats with PCCs and CCCs ranging from 0.68 to 0.94 and 0.63 to 0.94, respectively. Lower prediction performance was observed for the N-HP-LC habitat with a PCC and CCC of 0.77 and 0.73, respectively. Similarly, for the nodal lesion (Fig. 3b), strong correlation and agreement was observed for the H-LP-HC, H-HP-LC, and N-LP-HC habitats with PCCs and CCCs both ranging from 0.55 to 0.94. Again, lower prediction performance was observed for the N-HP-LC habitat with a PCC and CCC of 0.59 and 0.56, respectively. Figure 3c, d show habitat time courses for a representative patient with a primary tumor (Case 16, Fig. 3c) and nodal lesion (Case 21, Fig. 3d), while Fig. 3e, f show the spatiotemporal overall of the measured habitats for Cases 16 and 21.

Figure 4 shows the results of the predictions made with a single parameter for both primary and node diseases. For the primary tumor (Fig. 4a), strong correlation and agreement was observed for the H-LP-HC, H-HP-LC, and N-LP-HC habitats with PCCs and CCCs greater than 0.76 and 0.74, respectively. Lower prediction performance was observed for the HP-LC habitat with a PCC and CCC of 0.73 and 0.64, respectively. Similarly for the nodal lesion (Fig. 4a), strong correlation and agreement was observed for the H-LP-HC, H-HP-LC, and LP-HC habitats with PCCs and CCCs greater than 0.70 and 0.69, respectively. As with the primary tumor, lower prediction performance was observed for the HP-LC habitat with a PCC and CCC of 0.55. Figure 4b-d show representative time courses for Case 29 that had both primary (Fig. 4b) and nodal (Fig. 4c) disease and the corresponding habitats overlayed on the anatomical image (Fig. 4d).

Table 2 summarizes the ability of our model to predict significant changes in the hypoxic volume at W2 and W4 of radiotherapy for the primary tumor, nodal lesion, and both. Strong prediction performance was observed for the primary tumor at W2 and W4 with AUCs greater than 0.83, sensitivity of 1.00, and specificity greater than 0.75. Conversely, lower prediction performance was observed at W2 in the nodal lesions with an AUC of 0.61, sensitivity of 0.88, and specificity of 0.43. When the primary tumor and nodal lesion outcomes are combined, similar AUCs (0.70 vs. 0.69) and sensitivities (0.92 and 1.00) were observed, while the specificity increased (0.47 vs. 0.62) from W2 to W4.

**Table 2:** Results of receiver operator characteristic curve analysis

		Measured response			Youden’s Index		
		Decreased	Otherwise	AUC	Sensitivity	Specificity	Cut-off
W2	Combined	15	13	0.70	0.92	0.47	−23.20
	Primary	8	5	0.83	1.00	0.75	−19.27
	Node	7	8	0.61	0.88	0.43	−23.20
W4	Combined	16	2	0.69	1.00	0.63	−34.68
	Primary	7	2	0.93	1.00	0.86	−34.68
	Node	9	0	ND*	ND	ND	ND

ND Only one response category is present, therefore, no ROC analysis was performed.

## Discussion

While HPV-positive oropharyngeal carcinomas are generally responsive to chemoradiation, long-term treatment-related side effects remain a significant concern—motivating interest in RT de-escalation strategies to reduce unwanted toxicity without compromising tumor control. Dose de-escalation can be approached in two main ways: a priori or anticipatory de-escalation^7,12,32^. For a priori de-escalation, the treatment is adapted based on biomarkers such as hypoxia status assessed before or during therapy. In anticipatory de-escalation, the treatment is adapted based on predictive models to guide treatment decisions prior to or early during therapy^33,34^. To enable anticipatory adaptation, we have developed a novel habitat-imaging-based system of coupled ODEs that describes the temporal dynamics of hypoxic and normoxic tumor sub-volumes over the course of chemoradiation. This modeling framework retains key features of intratumoral heterogeneity while avoiding the need for longitudinal image registration or computationally intensive simulations. Model predictions are enabled through each patient’s unique tumor composition prior to RT and cohort-specific parameterization for both primary tumors and nodal lesions. When applied to patients with HPV-positive oropharyngeal carcinomas, our model was able to accurately predict changes in hypoxia within the primary tumor at weeks 2 and 4 of treatment, achieving area under the curve (AUC) values exceeding 0.83—highlighting its potential utility in guiding personalized de-escalation strategies.

Four habitats were selected reflecting variations in hypoxia, perfusion, and cellularity. These habitats were spatially contiguous and were well represented across all patients, tumor types, and imaging time points. While the number of habitats could be expanded (or reduced), using four habitats offers a balance between capturing intratumoral heterogeneity and enabling simplification of model outputs—such as total tumor volume or binary categories like hypoxic vs. normoxic, well-perfused vs. poorly perfused, and high vs. low cellularity. Expanding the number of habitats would require additional model parameters and introduce more assumptions about the biological interactions between compartments. As a first step toward model refinement, sensitivity analysis and parameter identifiability techniques could be applied to determine which additional parameters are necessary and justifiable within a more complex framework. In addition, model selection methods such as the Akaike Bayesian information criterion could be applied to a family of mathematical models built on a varying number of habitats and model parameters. This would provide a rigorous, data-driven framework to systematically identify the optimal level of model complexity (and the number of habitats) required to yield accurate predictions of specific quantities of interest while avoiding overfitting. The identified habitats may be specific to our dataset and tumor type; therefore, applying habitat-imaging-based modeling to other disease sites or datasets should begin with careful interpretation of the derived image-based habitats and selection of biologically meaningful compartments for model development. For example, ref. ^35^ identified four habitats in a murine model of breast cancer using DWI, DCE-MRI, *T*_*2*_ maps, and *T*_2_^*^ maps, validated against histological sections. Their habitats reflected varying levels of hypoxia, perfusion, and cellularity, and included: viable tissue (normoxic, high perfusion, and low cellularity), hypoxic viable (hypoxic, moderate perfusion, and high cellularity), hypoxic non-viable (hypoxic, low perfusion, high cellularity), and non-viable (low-perfusion, high cellularity). While these categories do not align exactly with the four habitats in our dataset, there is notable overlap in the underlying physiological features captured. Similarly, in our prior work^14,15^ (which did not incorporate OE-MRI for assessing hypoxia), we identified three habitats based on combinations of perfusion and cellularity: high perfusion and high cellularity, low perfusion and high cellularity, and low perfusion and low cellularity. Differences in habitat composition across studies could reflect variations in the underlying tumor biology (e.g., extent and prevalence of necrosis, degree of vascularization, size at detection or treatment, preclinical vs. clinical disease) and imaging methodology. For example, both refs. ^15,35^. employed DWI to estimate cellularity in murine models, while we employed *v*_*e*_ in the clinical setting to provide a surrogate measure of cellularity. Even though DWI and *v*_*e*_ are well correlated with cellularity^13,36,37^, the use of DWI instead of *v*_*e*_ could result in alternative habitat classifications. Ultimately, habitat-imaging-based modeling represents a high-level abstraction of the sub-voxel physiology^35^, and differences in imaging modality, voxel resolution, or tumor type can impact the resulting habitat definitions.

Building on prior work in the field^4,15^, we developed a four-compartment model to characterize tumor dynamics and response to RT, using a simplified but physiologically informed representation of tumor habitats. Our framework extends the model proposed by ref. ^15^, incorporating both the distinct imaging-derived habitats identified in our data and the delivery of RT. While ref. ^15^ used model selection techniques to reduce model complexity; we retained the full model structure to better capture variations across individual primary tumors and nodal lesions. As shown in Table 1, several parameters (*k*_*1*_, *k*_*2*_, *k*_*43*_, *k*_*41*_) could be eliminated in a cohort-level model due to limited impact on the overall dynamics. However, these parameters may be necessary to improve patient-specific predictions, particularly in cases where there are discordant growth or response patterns between primary and nodal disease. In such scenarios, individualized parameterization could improve model predictions over generalized cohort-derived parameters. Interestingly, our estimated RT efficacy parameter (*α*) ranged from 0.009 to 0.013 Gy^-1^, which lies at the lower end of values reported for head and neck cancers^38^ (0.001 to 0.584 Gy^−1^) and in previous modeling studies^39^ (0.090 Gy^−1^). To our knowledge, this study provides the first estimates of the model parameters *k*_*reoxy*_, *k*_*34*_, and *k*_*12*_, which correspond to reoxygenation and reperfusion in tumors, which are key radiobiological processes^40–43^ influencing treatment response. These parameters may help inform the timing and dosing of RT to exploit windows of increased tumor sensitivity to RT, particularly when the tumor is well-oxygenated or well-perfused. Notably, we observed that the tumors appear to have a higher reperfusion rate relative to their reoxygenation rate (i.e., higher *k*_*34*_ relative to *k*_reoxy_) following RT. Stratifying patients based on reperfusion rates may offer a valuable approach for guiding therapy, as previous studies in head and neck cancer have shown that increases in tumor perfusion following RT is correlated with improved patient outcomes^44–46^. More specifically, the prediction of a tumor’s habitat composition at weeks 2 or 4 during RT may provide a clinically meaningful opportunity to adapt treatment strategies. By monitoring changes in perfusion and oxygenation over time, clinicians could potentially identify patients who may benefit from modified dosing schedules or additional interventions aimed at mitigating toxicity. However, a larger study with increased cohort size and longer clinical follow-up is necessary to identify and validate the optimal thresholds for integrating modeling into clinical decision-making. Despite these current limitations, the feasibility of this approach is supported by recent work where pretreatment modeling parameters were successfully utilized to personalize RT fractionation^47^.

There are several opportunities to enhance our current modeling framework to address limitations in its predictions and improve clinical utility. First, to address inter- and intra-patient variations in tumor growth and response dynamics, future iterations of this approach could integrate data assimilation techniques^48–50^ to dynamically update model parameters as new data become available during therapy. For example, an initial prediction could inform a personalized baseline RT plan, which could be continued or refined during treatment (e.g., W2) based on new imaging data. This adaptive approach may improve prediction accuracy and allow for mid-treatment plan adjustments to maintain optimal tumor control. This personalized approach may improve accuracy, specifically addressing challenges where variations in individual nodal dynamics resulted in decreased predictive accuracy. Furthermore, while no statistically significant differences were observed within our current cohort regarding treatment sub-groups (Supplemental Table 1), this personalized approach would be particularly valuable for patients receiving either radiotherapy alone or combined chemo-radiotherapy, where distinct growth and response characteristics are likely to occur. In addition to stratifying patients based on treatment group, patients could be stratified based on habitat imaging “phenotypes”^14^, which are determined through a secondary clustering of the patient’s pretreatment habitat composition. This approach may provide a more granular understanding of a patient’s baseline status (in both primary and nodal disease) and could potentially identify cohorts that exhibit distinct response trajectories.

Second, optimal control theory approaches could enable the model to identify patient-specific RT strategies that balance tumor control with constraints on dose, delivery frequency, and normal tissue toxicity. Such approaches have been applied in the context of chemotherapy^51–53^ and RT planning^4,34,54^, and could be tailored to this model’s structure to improve clinical utility. However, while optimal control theory offers a mathematical pathway to dose personalization, the more immediate clinical strength of our modeling framework may lie in the potential to stratify patients among existing (and approved) treatment regimens. Rather than personalizing the dose every day, tumor habitat composition or tumor dynamics could be used as a decision support tool to determine which patients would benefit from dose escalation or dose deescalation. A similar approach was employed by ref. ^47^ in head and neck cancer, demonstrating the feasibility of integrating modeling in clinical workflows.

Both data assimilation and personalized treatment can be unified under a digital twin framework, such as that proposed by ref. ^34^, which emphasizes the importance of uncertainty quantification. Quantifying uncertainty in both model predictions and underlying measurements is essential for ensuring the safety and trustworthiness of digital twins in oncology^55,56^. We note that although our current framework does not explicitly quantify uncertainty in the model or measurement, this could be addressed through a Bayesian framework to infer the joint posterior distribution of model parameters and data.

While our current model operates at the tumor and sub-tumor scale, future work could consider spatially resolved models that describe the spatiotemporal dynamics of tumor habitats using partial differential equations. This approach could provide a more detailed representation of evolving tumor habitats and potentially enable adaptive, dose-painting strategies escalating (or de-escalating) radiation to specific sub-volumes—improving tumor control while minimizing treatment-related side effects. Finally, a future direction for this work would be to correlate imaging and modeling-based biomarkers with clinical endpoints (e.g., locoregional control and survival). Given the high cure rates and extended follow-up duration typically required for HPV-positive oropharyngeal cancers^57^, such clinical correlation was beyond the scope of this initial model development. However, ongoing longitudinal tracking of this cohort could be used to determine if the predicted habitat dynamics serve as surrogate markers for long-term treatment success.

## Methods

### Patient cohort

The salient details of the patient cohort and MRI acquisition are reported here, while complete details can be found in ref. ^20^. Patients (*N* = 20) with HPV-associated oropharyngeal carcinomas were recruited and consented to participate in a prospective clinical trial (NCT03646747) approved by a local institutional review board (REC 18/NW/0563) in accordance with the Declaration of Helsinki. Patients were imaged up to four times before and during RT on either a 1.5T diagnostic MR (Philips Ingenia MR-RT, Philips Medical Systems, Best) or a 1.5T MR Linac system (Elekta Unity, Elekta, Stockholm). The MRI protocol generally consisted of two pre-RT imaging visits (baseline 0 and 1, BL0 and BL1) separated by one week and two during RT (weeks 2 and 4; henceforth denoted as W2 and W4, respectively). All patients received RT between 55 Gy in 20 fractions to 70 Gy in 35 fractions, while a subset of patients (*N* = 12) received concurrent platinum-based therapy.

**Fig. 5:**
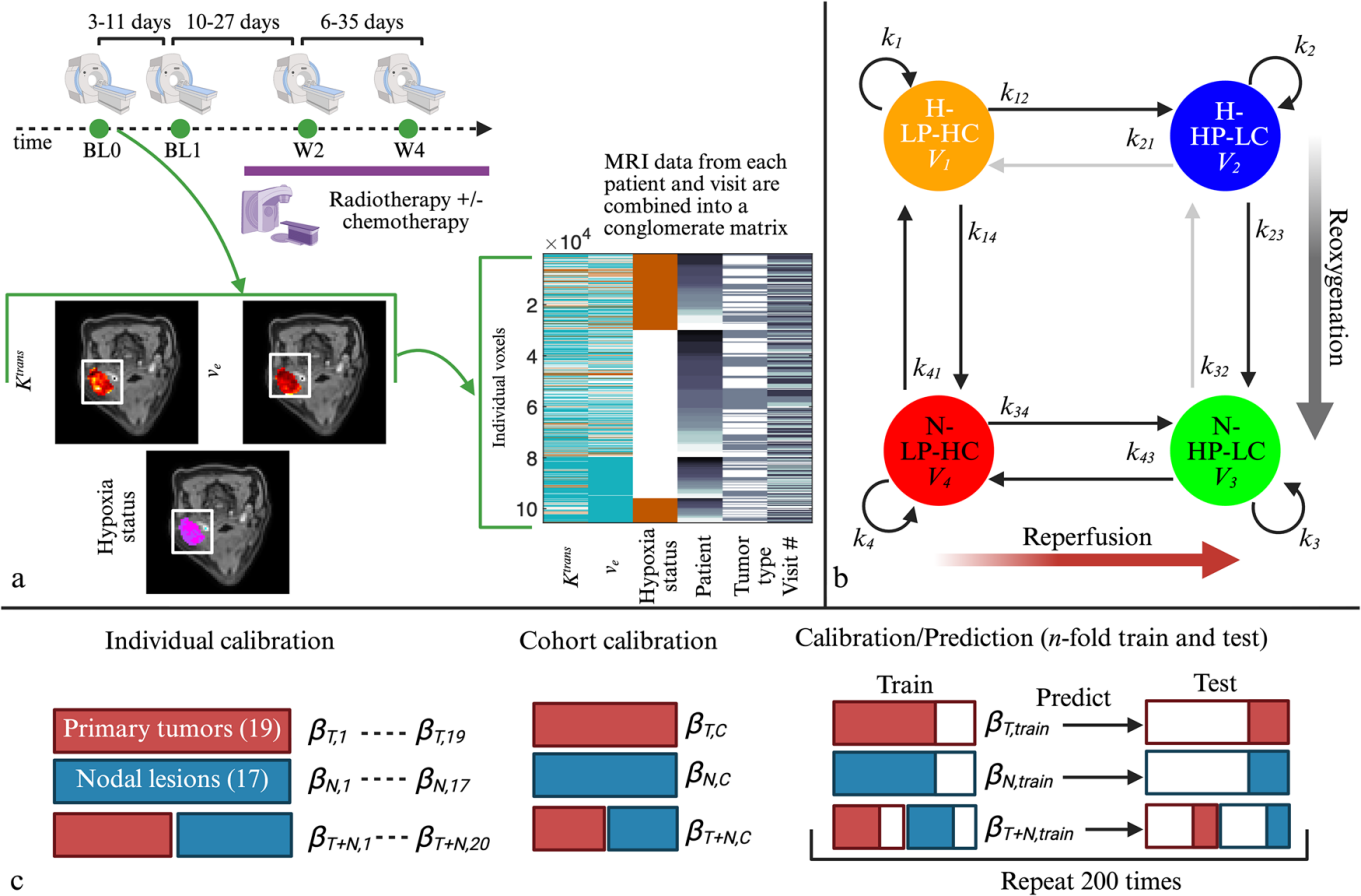
Schematic of image processing, modeling and calibration approaches. **a** Each patient undergoes up to four imaging sessions, during which DCE-MRI and OE-MRI are acquired to generate maps of *K*^trans^, *v*_*e*_, and hypoxia status. Voxel-wise values for these parameters are extracted from the primary tumor and nodal lesion regions of interest and aggregated into a composite matrix. This process is repeated for each patient and imaging visit. **b** A four-compartment mathematical model was developed to describe the change in hypoxic (H-LP-HC and H-HP-LC) and normoxic (N-LP-HC and N-HP-LC) habitat volumes. Each habitat has a growth rate (i.e., *k*_*i*_) and transfer rates between habitats (i.e., *k*_*ij*_). Movement from left to right represents reperfusion of the tissue, while movement from top to bottom represents reoxygenation. Grayed-out arrows indicate parameters removed from the full model. **c** Three calibration scenarios were conducted. First, the model was calibrated individually using each patient’s data for the primary, nodal, and combined (i.e., both primary and nodal) lesions. Second, cohort-wide calibration was performed using all patients. Finally, an *n*-fold calibration/prediction approach was implemented using 75% of the patients for cohort calibration and the remaining 25% for evaluating predictions. This approach was also applied to the primary, nodal, and combined tumor volumes. Created in https://BioRender.com.

At each imaging visit, dynamic OE-MRI and DCE-MRI were acquired and then analyzed using standard approaches^20^. OE-MRI data were analyzed to classify voxels as hypoxic (voxels that enhanced on DCE-MRI, but not on OE-MRI), normoxic (voxels that enhanced on both DCE-MRI and OE-MRI), or non-perfused (voxels that did not enhance on DCE-MRI). For the subsequent analysis, we excluded all non-perfused voxels (1.64% of the data). Enhancement for both DCE-MRI and OE-MRI was defined as voxels that had a statistically significant increase in signal intensity (*p* < 0.05) relative to the pre-gas challenge (for OE-MRI) or pre-contrast (for DCE-MRI) images. DCE-MRI data were analyzed with the standard Tofts model^44^ to yield maps of tissue perfusion (*K*^trans^) and extracellular volume fraction (*v*_*e*_). Primary tumors (T) and nodal lesions (N) were manually segmented on post-contrast *T*_*1*_-weighted images by a head and neck oncologist (7 years’ experience) using JIM software (JIM 6, Xinapse Systems, RRID: SCR_009589). Image processing and analysis were performed using MATLAB (R2018a, MathWorks, Natick, MA). All images were registered and resampled to have voxel dimensions of 3 × 3 × 2.5 mm^3^. Table 3 summarizes the clinical information for the patients used in the subsequent analysis, while Fig. 5a shows the experimental timeline. Patient identification numbers are non-sequential to maintain consistency with previous publications^20^. Exclusions from the original cohort (i.e., IDs omitted in Table 3) were due to a lack of measurable disease on MRI, protocol deviations at baseline, technical failures during gas inhalation, or the absence of longitudinal follow-up imaging.

**Table 3:**
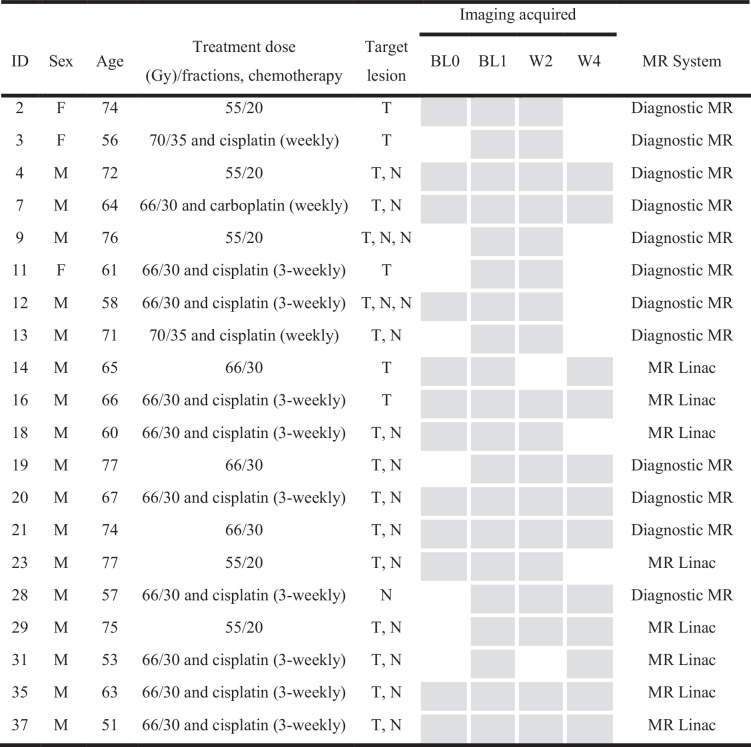
Clinical and imaging information of the patient cohort

### Habitat identification

The quantitative image analysis yielded a three-dimensional vector of MRI parameters (*K*^trans^, *v*_*e*_, and tissue oxygenation status) at each voxel within the T and N regions of interest of each patient at every imaging timepoint. All three-dimensional vectors were pooled into one matrix where each column of the matrix represents a different MRI parameter, and each row represents information for a specific voxel from a particular patient and imaging timepoint (as shown in Fig. 5a). As *v*_*e*_ is defined as a volume fraction, it is bounded within [0, 1] during image analysis. Likewise, *K*^trans^ was constrained to be greater than 0. This ensures that the parameter estimates remain physically meaningful and prevents outliers from propagating through the subsequent analysis. Tissue oxygenation status was treated as a categorical variable, and assigned values of −2 for hypoxic or 2 for normoxic voxels. This magnitude was determined empirically by iteratively increasing the value until the clustering algorithm consistently achieved separation of habitats based on oxygenation status, thereby preventing the grouping of hypoxic and normoxic regions into the same cluster. While an alternative approach would involve clustering hypoxic and normoxic voxels separately, we elected to process the complete dataset simultaneously to identify habitats based on the full range of physiological parameters. We then employed *k*-means clustering^14,15,23^ to identify tumor habitats in terms of the level of perfusion (characterized by *K*^trans^), cellularity (characterized by *v*_*e*_), and oxygenation status (characterized by OE-MRI). Elbow curve analysis (see Supplemental Material S1 for details) was employed to help select the optimal number of tumor habitats, which was found to be four. After the selection of the optimal number of habitats, we interpreted the habitats as “normoxic high-perfusion low-cellularity” (N-HP-LC), “normoxic low-perfusion high-cellularity” (N-LP-HC), “hypoxic high-perfusion low-cellularity” (H-HP-LC), and “hypoxic low-perfusion high-cellularity” (H-LP-HC) based on the relative mean values of the centroids. While no spatial information was included in the clustering, multiregional spatial interaction matrix analysis^24^ verified that the identified habitats were spatially co-localized (see Supplemental Material S2 for details). The result of the habitat analysis for a given patient and tumor is a set of time courses for each tumor habitat.

### Mathematical model of tumor growth and response to RT

Figure 5b and Eqs. (1–4) present a four-compartment model describing the dynamics of each imaging-based habitat throughout RT. Equations (1–4) are composed identically, describing the change in the volume of habitat *i*, *V*_*i*_, due to proliferation *k*_*i*,_ transition to a different habitat *j* at rate *k*_*ij*_, or transition to habitat *i* from a different habitat *j* at rate *k*_*ji*_ :

1 $$\frac{d{V}_{1}}{{dt}}={V}_{1}(t)\left({k}_{1}-{k}_{12}-{k}_{14}(t)\right)+{V}_{2}(t){k}_{21}+{V}_{4}(t){k}_{41},$$

2 $$\frac{d{V}_{2}(t)}{{dt}}={V}_{2}(t)\left({k}_{2}-{k}_{21}-{k}_{23}(t)\right)+{V}_{1}{(t)k}_{12}+{V}_{3}{(t)k}_{32},$$

3 $$\frac{d{V}_{3(t)}}{{dt}}={V}_{3}(t)\left({k}_{3}-{k}_{32}-{k}_{34}\right)+{V}_{2}{\left(t\right)k}_{23}(t)+{V}_{4}{(t)k}_{43},$$

4 $$\frac{d{V}_{4}(t)}{{dt}}={V}_{4}\left(t\right)\left({k}_{4}-{k}_{41}-{k}_{43}\right)+{V}_{1}{\left(t\right)k}_{14}(t)+{V}_{3}{\left(t\right)k}_{34}.$$

Response to RT is modeled using the linear-quadratic^58^ model, shown in Eq. (5), to determine the tumor volume of habitat *i* immediately after RT, *V*_*i*,post,_ given the tumor volume immediately before RT*, V*_*i,*pre_, radiosensitivity parameters *α* and *β*, and RT dose per fraction ${Dose}\left(t\right)$.

5 $${V}_{i,{post}}(t)={V}_{i,{pre}}(t){e}^{-\alpha {Dose}\left(t\right)-\beta {{Dose}(t)}^{2}}$$

Equation (5) describes an immediate reduction in the tumor volume after the delivery of a single fraction of RT.

Several of the model parameters can be interpreted in terms of the “Rs” of radiobiology^40–43^ as follows: the *k*_*i*_ terms represent repopulation, *k*_*14*_ and *k*_*23*_ represent reoxygenation, and *k*_*21*_ and *k*_*43*_ represent reperfusion or remodeling of the tumor microenvironment. To reduce the number of unknown parameters, we assume that highly perfused, hypoxic tissues cannot transition to low-perfusion hypoxic tissues (*k*_*21*_ = 0) and that highly perfused normoxic tissues do not transition to highly perfused hypoxic tissues (*k*_*32*_ = 0). Furthermore, we assume that the reoxygenation rates are equal (*k*_*14*_ = *k*_*23*_) and linearly increase with each fraction of RT as in Eq. (6):

6 $${k}_{14}\left(t\right)\equiv {k}_{23}\left(t\right)={k}_{{reoxy}}\frac{{\sum }_{i=1}^{t}{Dose}\left(t\right)}{{{Dose}}_{{total}}}$$

where *k*_reoxy_ is the reoxygenation rate, and Dose_total_ is the total radiation dose delivered. We assign the *α*/*β* ratio of 10 Gy from literature^38,39^ and assume *α* for hypoxic tissue is equal to *α*/1.5^59,60^. The model were implemented and solved in MATLAB (R2024b, MathWorks, Natick, MA) using a forward Euler finite difference approximation with a time step of 0.01 days. Table 4 summarizes the model variables and parameters.

**Table 4:** Description of model parameters and variables

Parameter or variable	Description	Assignment or bounds for calibration	
*V*_*1*_	H-LP-HC habitat volume	Assigned from habitat imaging (cm^3^)	
*V*_*2*_	H-HP-LC habitat volume	Assigned from habitat imaging (cm^3^)	
*V*_*3*_	N-HP-LC habitat volume	Assigned from habitat imaging (cm^3^)	
*V*_*4*_	N-LP-HC habitat volume	Assigned from habitat imaging (cm^3^)	
*Dose*(*t*)	Dose of RT per fraction	Assigned from patient prescription (Gy)	
*β*	Radiosensitivity parameter	Calculated assuming *α/β* of 10 Gy^39^	
		Lower bound	Upper bound
*k*_*i*_	Proliferation rate	0 day^−1^	2.5 day^−1^
*k*_*ij*_	Transition rates from habitat *i* to *j*	0 day^−1^	2.5 day^−1^
*k*_*reoxy*_	Reoxygenation rate	0 day^−1^	2.5 day^−1^
*α*	Radiosensitivity parameter	0 Gy^−1^	0.05 Gy^−1^

### Calibration and prediction scenarios

A total of ten parameters were calibrated from the patient data, including four proliferation rates, four transition rates, *k*_reoxy_, and *α*. We defined the set of parameters as *β*_*T*_ for the primary tumor, *β*_*N*_ for the nodal lesion, and *β*_*T+N*_ for the combined primary and nodal lesions. *β*_*T*,_ *β*_*N*,_ and *β*_*T+N*_ were calibrated through three distinct calibration scenarios as shown in Fig. 5c. First, we performed individual calibrations for each primary tumor (19 tumors; *β*_*T,1*_ to *β*_*T,19*_), nodal lesion (17 lesions; *β*_*N,1*_ to *β*_*N,17*_), and combined primary and nodal lesions (20 cases; *β*_*T+N,1*_ to *β*_*T+N,20*_). Second, we conducted cohort-wise calibrations to determine a single optimal parameter set representing the entire cohort: *β*_*T,C*_, *β*_*N,C*_, and *β*_*T+N,C*_. Finally, we implemented an *n*-fold calibration and prediction scenario where *β*_*T,train*_, *β*_*N,train*_, and *β*_*T+N,train*_ were estimated from a training subset of patients (75% of the patients) and used to predict responses in a held-out subset. This *n*-fold process was repeated 200 times to ensure at least 50 unique predictions per patient.

To reduce the risk of convergence to local minima, each calibration followed a multi-step procedure. First, a global search was performed using particle swarm optimization (*particleswarm* in MATLAB). This was followed by local refinement using nonlinear least squares optimization (*lsqnonlin* in MATLAB) with the trust-region reflective method. Parameters were bounded as shown in Table 4, and the residual was defined as the difference between model-estimated and observed volume for each habitat and at each available time point.

For the prediction scenario, the model was initialized with each patient’s baseline habitat composition and the parameter set (*β*_*T,*train_, *β*_*N,*train_, or *β*_*T+N,*train_) calibrated from the training cohort. All calibrations and predictions were performed on a personal computer equipped with an Apple M1Max (3.2 GHz, ten cores) and 64 GB of memory. To accommodate irregularly sampled data for the model calibration and prediction scenarios, the model was evaluated only at the specific time points indicated in Table 3 for each patient. Supplemental Material S3 reports sensitivity and parameter identifiability analysis results for this modeling framework.

### Statistical analysis

The Pearson correlation coefficient (PCC) and Lin’s concordance correlation coefficient^61^ (CCC) were used to assess the correlation and agreement, respectively, between observed and model-estimated tumor volumes for each habitat. For individual patient predictions, the average of 50 predictions was used. When appropriate, statistics were reported as mean and standard deviation if they were normally distributed (determined using the Lilliefors test; *lillietest* in MATLAB), or median and range otherwise.

For the prediction scenario, receiver operator characteristic (ROC) analysis was used to evaluate the model’s ability to predict significant changes in hypoxic volumes at W2 and W4 in both the primary and nodal lesions. A significant change was defined as one that fell outside the repeatability bounds (−45.7 to 84.1%)^20^. ROC analysis provided the area under the curve (AUC) based on Youden’s index, as well as the corresponding sensitivity, specificity, and optimal cutoff (i.e., the threshold applied to model predictions to indicate a change in hypoxic volume).

## Supplementary information

Supplementary information

## Data Availability

The raw data that support the findings of this study (i.e., medical imaging data and resulting habitat time) are not openly available due to restrictions that apply to the availability of these data, but the data may be requested from the corresponding author upon reasonable request.

## Code availability

Code and publicly available data to recreate the key figures are shared at 10.6084/m9.figshare.30113692.
