# Supplementary material for: Predicting head and neck cancer response to radiotherapy using mathematical modeling of MRI-based habitats
Source: NPJ Precis Oncol. 2026 Apr 16;10:276. doi: 10.1038/s41698-026-01344-x (PMC13357755; doi:10.1038/s41698-026-01344-x)
Supplement: Supplementary file 1 — Supplementary information [file 41698_2026_1344_MOESM1_ESM.pdf]

Supplemental material:

S.1 Elbow analysis for identifying optimal number of tumor habitats

Elbow analysis determines the optimal number of clusters or components for clustering methods such as k-means. The selected number of tumor habitats reflected the number of clusters which best separated meaningful differences amongst the three MRI parameters of each voxel. We performed an elbow analysis on the calculated within-cluster sum of squares (WCSS(i)) for 1 to 10 clusters shown in **Supplemental Figure 1**. The WCSS quantifies how close data points are grouped around their cluster centroids for that number of clusters. As the number of clusters increased, the WCSS decreased as points are placed into smaller and more homogenous groups. The "elbow" at 4-6 clusters in the WCSS curve indicated adding more clusters beyond that point captured noise instead of meaningful structure making 4-6 clusters the optimal number. We chose to proceed with four clusters which provided consistent and interpretable habitat definitions across hypoxic and normoxic tumor habitats.

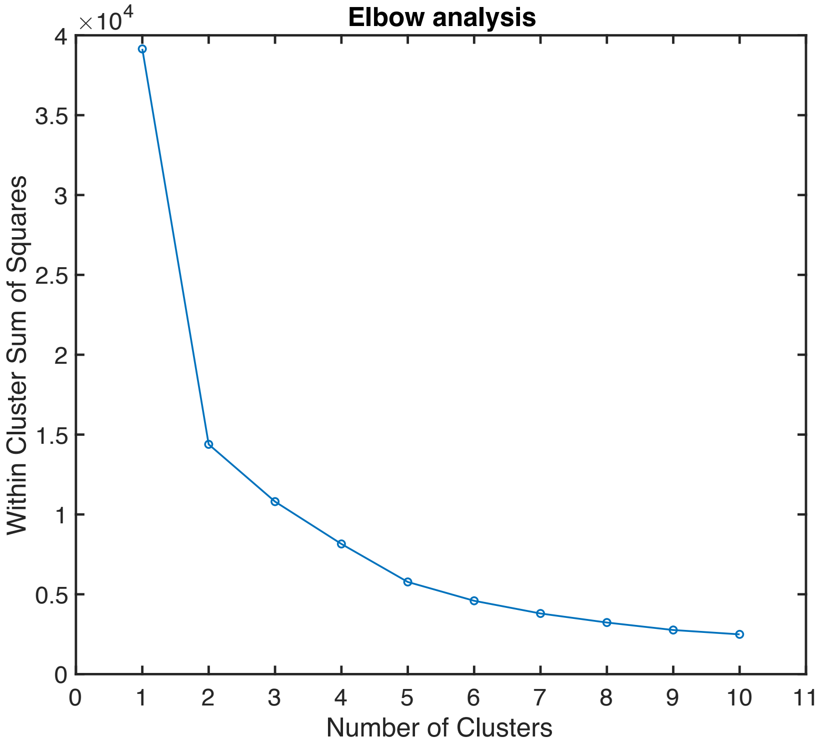

Supplemental Figure 1: Elbow analysis to determine optimal number of clusters. Within cluster sum of square errors are reported as a function of the number of clusters.

S.2. Multiregional spatial interaction analysis

Multiregional spatial interaction (MSI) analysis (2) determines the spatial colocalization of habitat voxels by comparing the spatial correlation for the original mapping (i.e., that assigned by habitat imaging) versus randomly assigned mapping. Briefly, different combinations of voxel-neighbor pairs (e.g., H-LP-HC, H-HP-LC, HP-LC, LP-HC) were organized as a 4 × 4 matrix referred to as the MSI matrix. We then iterated through all tumor voxels and tallied their interactions with each neighbor. The diagonal elements of the MSI matrix correspond to connections between the same habitat type. We normalized this value by the total number of voxels to return the normalized MSI value. We perform this analysis for both the original mapping and a randomly assigned mapping. **Supplemental Figure 2** shows an example of one tumor's original and randomly assigned tumor habitats (panel **a**) and then the MSI calculated across the cohort (panel **b**). Note, that for all habitats the original mapping achieves a higher MSI relative to the random mapping indicating greater spatial co-localization of tumor habitats.

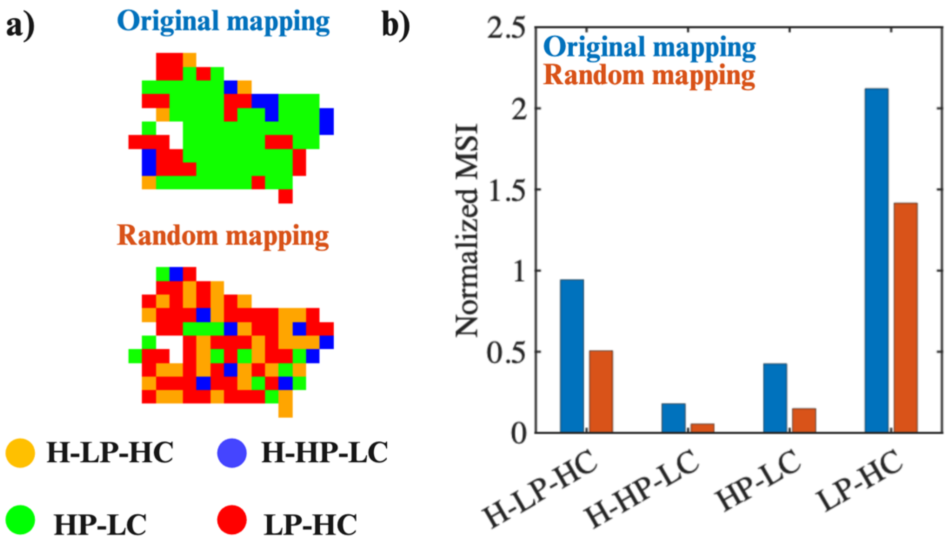

Supplemental Figure 2. Multiregional spatial interaction analysis. Panel **a)** shows the original mapping (as assigned via habitat imaging) and random mapping of tumor habitats for a given tumor. Panel **b)** reports the normalized MSI for each habitat. A larger normalized MSI was observed for each habitat indicating a greater degree of co-localization over randomly assigned habitat values.

S.3. Sensitivity and identifiability analysis

A sensitivity analysis using Sobol's method (3) was applied to the model for all patients using each patient's initial conditions, imaging schedule, and treatment schedule. We focused primarily on the total effect indices that identify the importance of a given parameter on a model output including secondary effects from parameter interactions. Model parameters were assumed to have uniform distributions within the bounds observed from the individual calibration (Table 3). A sample size of 5000 was selected to ensure convergence of the indices, while our quantity of interest (QoI) for sensitivity testing was defined as the habitat volumes for visits BL2, W2, and W4 and the total tumor volume. The Sobol' total effect indices (which are normalized measures relative to the output variation due to each parameter) are shown below (**Supplemental Figures 3-4**) for each model parameter and quantity of interest. The total effect indices indicated k_{43}, $k_{12,}$ and α were highly sensitive parameters for multiple QoI, while k_{reoxy} had total effect indices less than 0.1 for all QoI.

We further evaluated identifiability by subjecting the model to synthetic data corrupted with Gaussian noise ranging from 1% to 50%. These results (Supplemental Figure 5) demonstrate that most parameters have less than 20% error at moderate noise levels (10–20%), though identifiability generally decreases as noise approaches 50%.

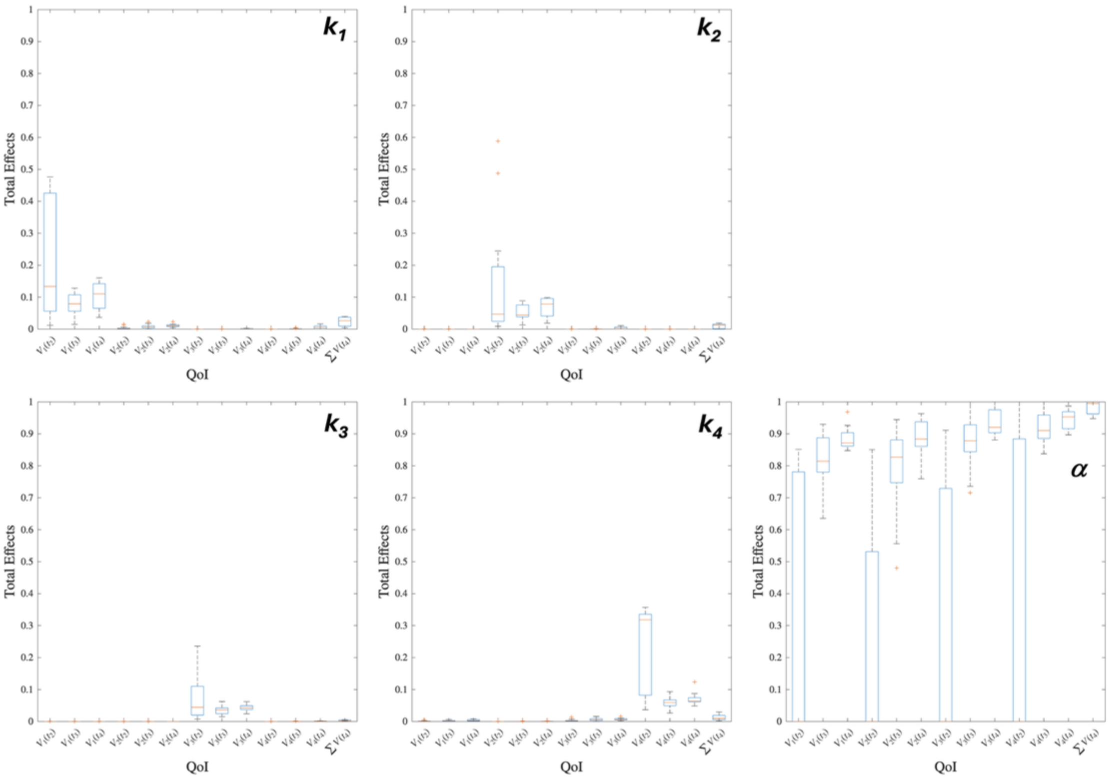

Supplemental Figure 3: Total Sobol' indices for k_1, k_2, k_3, k_4, and α for all quantities of interest. The sensitivity analysis indicates α is a highly sensitive parameter for multiple QoI, while the other parameters showed moderate sensitives (total effect indices > 0.1) for all QoI.

We further evaluated identifiability by subjecting the model to synthetic data corrupted with Gaussian noise ranging from 1% to 50%. These results demonstrate that most parameters have less than 20% error at moderate noise levels (10–20%), though identifiability generally decreases as noise approaches 50%.

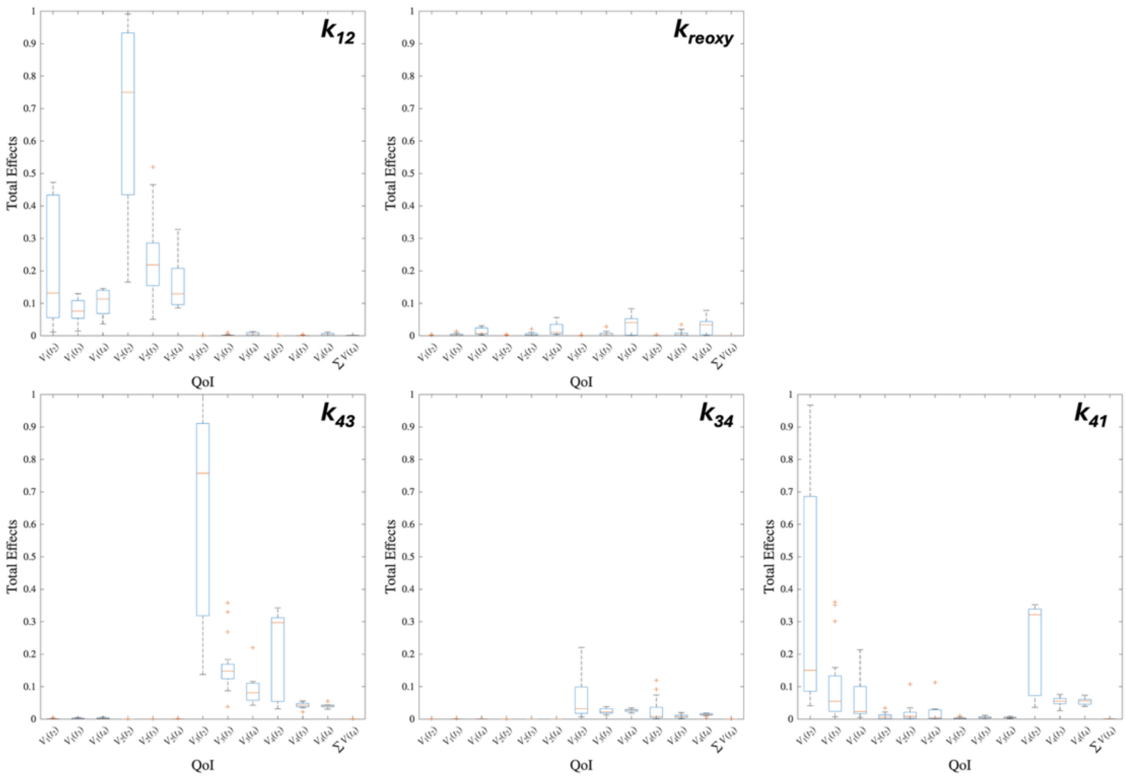

Supplemental Figure 4: Total Sobol' indices for k_{12}, k_{reoxy}, k_{43}, k_{34}, and k_{41} for all quantities of interest. The sensitivity analysis indicates that k_{43}, k_{41}, and k_{12} are highly sensitive parameters for multiple QoI, while k_{reoxy} had total effect indices < 0.1 for all QoI.

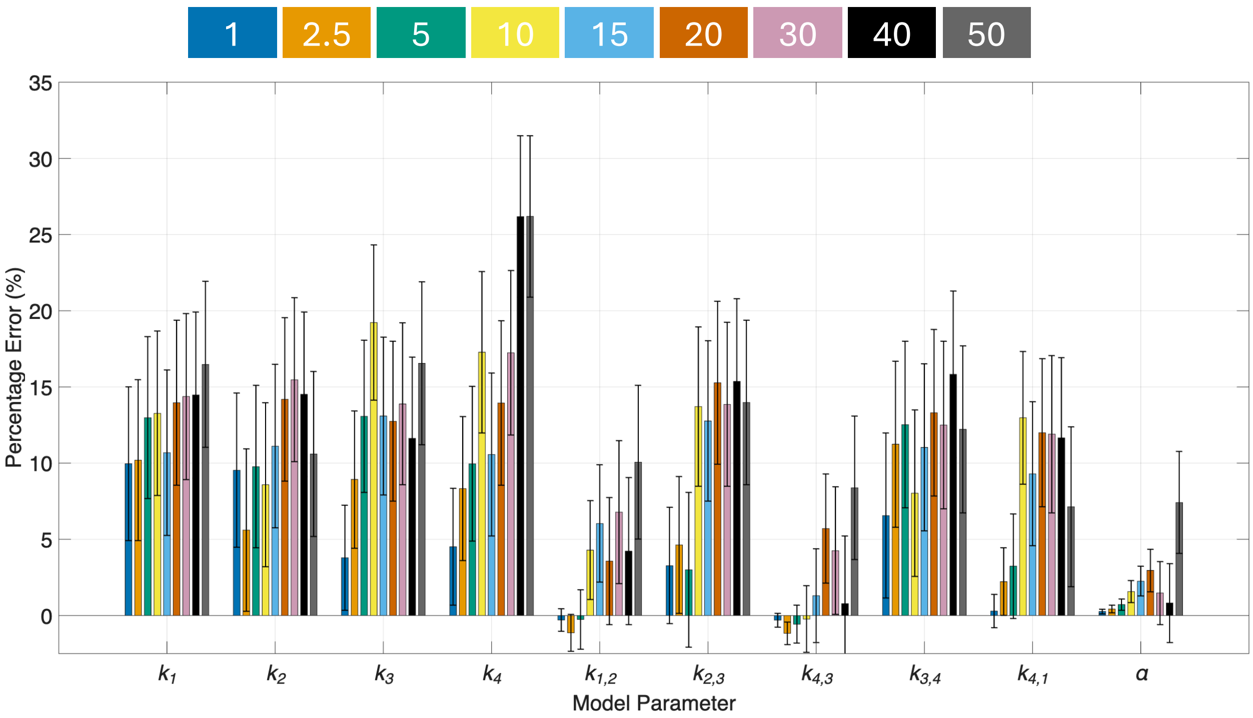

Supplemental Figure 5: Effect of measurement noise ranging from 1 to 50% on the identifiability of model parameters. For each parameter, the mean and standard deviation of the percentage error between the calibrated parameter and the ground truth parameter used for the synthetic dataset are reported.

S.4. Influence of MR system on habitat imaging clusters

To evaluate the influence of MR system (MR Linac vs. Diagnostic MR) we performed our habitat imaging analysis on three distinct groupings: the entire dataset, the MR Linac data alone, and the Diagnostic MR data alone. The mean and standard deviations for K^{trans} and v_e are shown in Supplemental Figure 3 for all three approaches. Our analysis showed no significant differences between the identified habitat centroids across these methods, confirming that the habitat definitions are consistent regardless of the specific MR hardware used for acquisition.

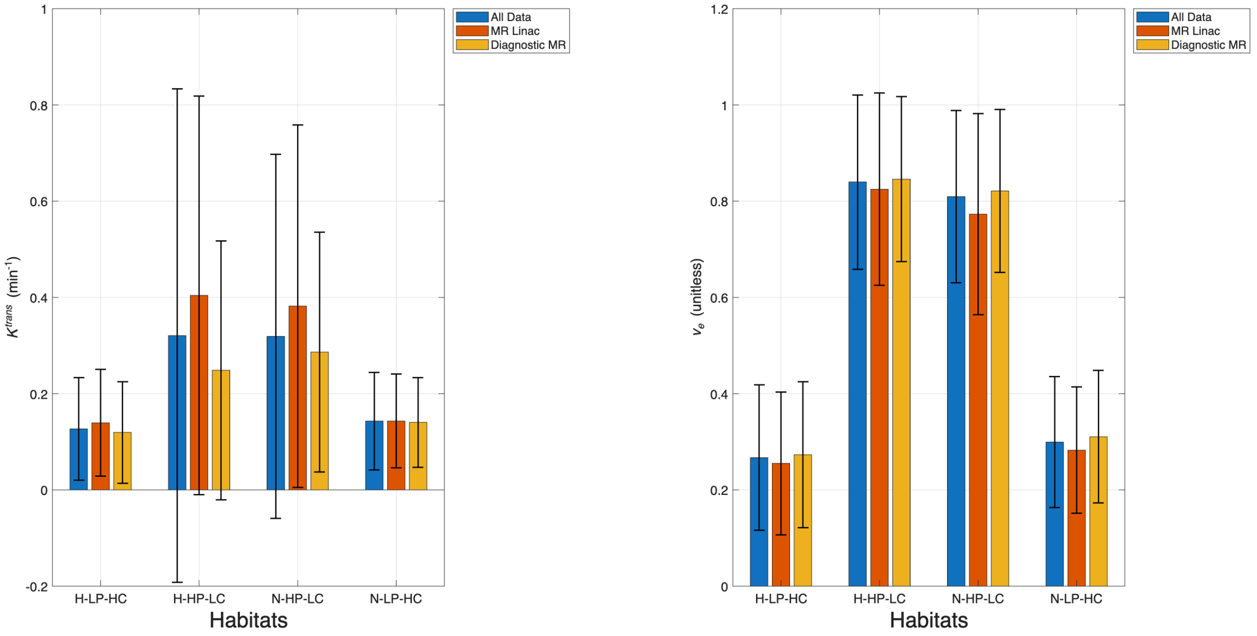

Supplemental Figure 6. We evaluated the influence of MR system on the resulting habitat clustering by performing habitat imaging either the entire dataset, MR Linac only, and Diagnostic MR only. The plots above show the mean and standard deviation for each habitat for K^{trans} (left plot) or v_e (right plot). No significant differences were observed between any of the subsets of data.

S.5 Calibrated model parameters across different treatment groups

To evaluate the impact of chemotherapy on the estimated kinetic parameters, we performed a comparative statistical analysis across three experimental conditions: Primary, Node, and Combined (Both). For each condition, individual subjects were categorized into two groups based on the presence or absence of chemotherapy. The median [minimum maximum] is reported for each parameter in Supplemental Table 1. Statistical differences between the groups for the 10 fitted parameters were assessed using the Wilcoxon Rank-Sum. A p-value of less than 0.05 was set as the threshold for statistical significance. No significant differences were observed between the chemotherapy and non-chemotherapy groups for any of the kinetic parameters across the primary, nodal, or combined datasets. All calculated p-values were greater than 0.075.

Supplemental Table 1. Calibrated model parameters reported by treatment group

		Primary	Node	Combined
		Individual	Individual	Individual
Patients with radiotherapy and chemotherapy	k_1 (day^{-1})	0 [0 0.108]	0 [0 0]	0 [0 .108]
	k_2 (day^{-1})	0 [0 0.171]	0 [0 0.014]	0 [0 0.199]
	k_3 (day^{-1})	0.035 [0 0.157]	0.052 [0 0.260]	0.116 [0 0.518]
	k_4 (day^{-1})	0.011 [0 0.185]	0.008 [0 0.162]	0.041 [0 0.242]
	k_{12} (day^{-1})	0 [0 0.540]	0 [0 0.158]	0 [0 0.537]
	k_{reoxy} (day^{-1})	0.003 [0 0.152]	0.012 [0 0.101]	0.044 [0 0.219]
	k_{34} (day^{-1})	0.167 [0 0.586]	0.259 [0 0.783]	0.537 [0 1.141]
	k_{43} (day^{-1})	0 [0 0.020]	0 [0 0.001]	0 [0 0.020]
	k_{41} (day^{-1})	0 [0 0.541]	0 [0 0]	0 [0 0.541]
	α (Gy^{-1})	0.008 [0 0.036]	0.010 [0 0.052]	0.004 [0 0.318]
Patients with only radiotherapy	k_1 (day^{-1})	0 [0 0.013]	0 [0 0.004]	0 [0 0]
	k_2 (day^{-1})	0 [0 0.532]	0 [0 0.199]	0 [0 0.048]
	k_3 (day^{-1})	0.105 [0.015 0.594]	0.066 [0.022 0.518]	0.067 [0.020 0.639]
	k_4 (day^{-1})	0.012 [0 0.183]	0.020 [0 0.242]	0.033 [0 0.137]
	k_{12} (day^{-1})	0.031 [0 0.561]	0.070 [0 0.522]	0.017 [0 0.502]
	k_{reoxy} (day^{-1})	0.039 [0 0.151]	0.032 [0.022 0.049]	0.033 [0.002 0.128]
	k_{34} (day^{-1})	0.470 [0.037 1.175]	0.188 [0.102 1.141]	0.360 [0.051 1.199]
	k_{43} (day^{-1})	0 [0 0]	0 [0 0]	0 [0 0]
	k_{41} (day^{-1})	0 [0 0.026]	0 [0 0.014]	0 [0 0.069]
	α (Gy^{-1})	0.004 [0 0.027]	0.006 [0 0.018]	0.010 [0 0.034]

References:

1. Brusco MJ, Steinley D. A Comparison of Heuristic Procedures for Minimum Within-Cluster Sums of Squares Partitioning. Psychometrika. 2007 Dec 1;72(4):583–600.

2. Wu J, Cao G, Sun X, Lee J, Rubin DL, Napel S, et al. Intratumoral spatial heterogeneity at perfusion MR imaging predicts recurrence-free survival in locally advanced breast cancer treated with neoadjuvant chemotherapy. Radiology. 2018;288(1):26–35.

3. Sobol′ IM. Global sensitivity indices for nonlinear mathematical models and their Monte Carlo estimates. Mathematics and Computers in Simulation. 2001;55(1):271–80.
